# Association of apolipoprotein B XbaI (rs693) polymorphism and gallstone disease risk based on a comprehensive analysis

**DOI:** 10.1186/s41021-021-00189-z

**Published:** 2021-05-03

**Authors:** Haifeng Zhu, Linhai Yu, Linsong Feng

**Affiliations:** Department of Surgery, Fangta Hospital of Traditional Chinese Medicine, Songjiang District, 201600 Shanghai, China

**Keywords:** Apolipoprotein B, Gallstone disease, Polymorphism, Meta‐analysis, Risk

## Abstract

**Background:**

Our aim was to investigate the association between XbaI gene polymorphisms in the apolipoprotein B (*APOB*) gene and gallstone disease (GD) risk through a comparison of the allele and genotype distribution frequencies at this site using meta-analysis.

**Methods:**

A literature search was performed using PubMed and Wanfang through Jun 1, 2020. Odds ratios (ORs) and 95 % confidence intervals (CIs) were used to assess the strength of associations.

**Results:**

After a comprehensive search, 14 different articles that met the inclusion criteria were selected, with 1583 cases and 1794 controls. Individuals carrying the A-allele or AA genotype of the rs693 polymorphism were determined to possibly have an increased risk of GD. For example, there was a significant relationship between the rs693 polymorphism and increased GD risk in the whole group (OR: 1.40, 95 % CI: 1.05–1.87 in the allelic contrast model), the Asian population (OR: 1.58, 95 % CI: 1.48–2.84 in the heterozygote model), and the hospital-based source of the control (OR: 1.79, 95 % CI: 1.13–2.84 in the dominant model).

**Conclusions:**

This study suggests that the *APOB* rs693 polymorphism is potentially associated with GD susceptibility, which might offer a detection marker for use in future large scale clinic research.

## Background

Gallbladder disease (GD) is a highly prevalent condition affecting up to 15 % of the population with a significant health care burden in the United States [1–3]. Approximately 10–20 % of the population will develop GD in their lifetime [4], and women are more than twice as likely as men to develop the disease [5]. Based on current information using ultrasound surveys, ethnicity is a known risk factor; specifically, the highest rate of GD is found in Hispanic people from central and south American heritage [2, 3]. The north Indian population also shows a high incidence of GD, affecting 64.1 % women and 29.5 % men [2]. On the other hand, individuals of African American, African, and East-South Asian (China, Japan, India, and Thailand) descent show lower incidence of GD development [6].

Besides race, there are many other factors for GD development, such as advanced age, sex, and a hypercaloric diet rich in carbohydrates and poor in fiber. Additionally, obesity is one of the most important predisposing factor for GD. Other factors that affect the hepatic production of cholesterol, stasis/inflammation, bile acid production, or intestinal absorption of cholesterol and bile acids also contribute to GD development. Increasing evidence also points to genetic factors as being important for GD development [4, 7].

The apolipoprotein B (*APOB*) gene, presumably affecting the lipid composition and lipid metabolism [8, 9], plays an important role in GD development. The XbaI polymorphism site (rs693) is located in exon 26 of the *APOB* gene [10, 11], which is a synonymous variant. It is well known that synonymous single-nucleotide polymorphisms (SNPs) are categorized as spurious events under no to modest selection through alterations to a nucleotide at a synonymous codon but retaining the encoded amino acid [12]. Synonymous SNPs are not randomly distributed across genes and preferentially target conserved sites [13]. In addition, synonymous mutations, which account for a larger proportion of somatic mutations detected in human pathology, play an important role in disease penetrance and are presumed to be driving mutations in some diseases [14], such as GD. The relationship between this polymorphism and GD has been examined in several studies; however, the conclusions have been unclear [15–28].

In a previous study, Niu et al. performed a meta-analysis and suggested that the rs693 polymorphism is significantly associated with higher levels of APOB, triglycerides (TG), total cholesterol (TC), and low-density lipoprotein cholesterol (LDL-C). Our current study comprised a similar meta-analysis, because GD is associated with the metabolism of TG, TC, and LDL-C [29]. To overcome factors such as sample size and regional and ethnic differences, our study summarized all published literature on the relationship between the XbaI polymorphism and GD based on meta-analysis, to comprehensively evaluate this relationship and provide an evidence-based medical basis for the etiology of GD.

## Materials and methods

### Literature search strategy

A computerized literature search was performed for relevant studies from PubMed and Wanfang published before Jun 1, 2020. The following keywords were jointly used: “Apolipoprotein B or *APOB* or Apo B,” “polymorphism or variation or mutation,” “rs693,” and “gallstone or cholelithiasis or biliary stone or bile duct stone.” If studies applied the same clinical case information, only the largest sample size was selected.

### Inclusion criteria

The included studies met the following criteria: (a) clear criteria for the diagnosis of GD, such as B-ultrasound, CT, MRI, or endoscopic retrograde cholangiopancreatography, among others; (b) a correlation between GD risk and *APOB* gene rs693 polymorphism; (c) case-control or cohort design; (d) providing sufficient data for calculating the odds ratio (OR) with a 95 % confidence interval (CI); (e) duplicate studies with the same cases; (f) the genotype distribution in the control group was in accordance with the Hardy-Weinberg equilibrium (HWE) law.

### Data extraction

The following information was extracted from each included study: name of the first author, publication year, country of origin, ethnicity, numbers of cases and controls, HWE of control group, genotyping method, and number of genotypes in cases and controls. The data were selected independently by two investigators who reached a consensus on all items.

### Statistical analysis

The associations between the *APOB* rs693 polymorphism and risk of GD were estimated by calculating the OR and 95 % CI. The statistical significance of the OR [30] and the significance of the effect for the correlation were determined using Z test. The heterogeneity among studies was evaluated using Q test and *I*^2^ test as described previously [31, 32]. As a guide, *I*^2^ values < 25 % might be considered “low,” a value of ~ 50 % might be considered “moderate,” and values > 75 % might be considered “high” [33]. The Mantel-Haenszel (fixed effect) model was chosen, and otherwise, if *P*_heterogeneity_ < 0.1, the random effects (DerSimonian-Laird) model was applied [34, 35]. Sensitivity analysis was undertaken by removing each study once to assess whether any single study could influence the stability of results [36]. The departure of frequencies of the rs11200638 polymorphism from expectation under the HWE was assessed using Pearson’s χ^2^ test, and *P* < 0.05 was considered significant [37]. Begg’s funnel plots and Egger’s regression test were performed to estimate the potential publication bias [38]. All statistical tests for this meta-analysis were performed using the Stata software, version 10.0 (StataCorp LP, College Station, TX, USA).

### Meta‐regression

Considering the subgroups of publication year, ethnicity, source of control as independent variables, and the log as a dependent variable, the random-effect meta-regression results were presented.

### Protein-interaction network of the *APOB* gene

To more completely understand the role of APOB in GD, the gene–gene interaction network for *APOB* was predicted using the online String database (http://string-db.org/) [39].

## Results

### Study search and basic information

**Fig. 1:**
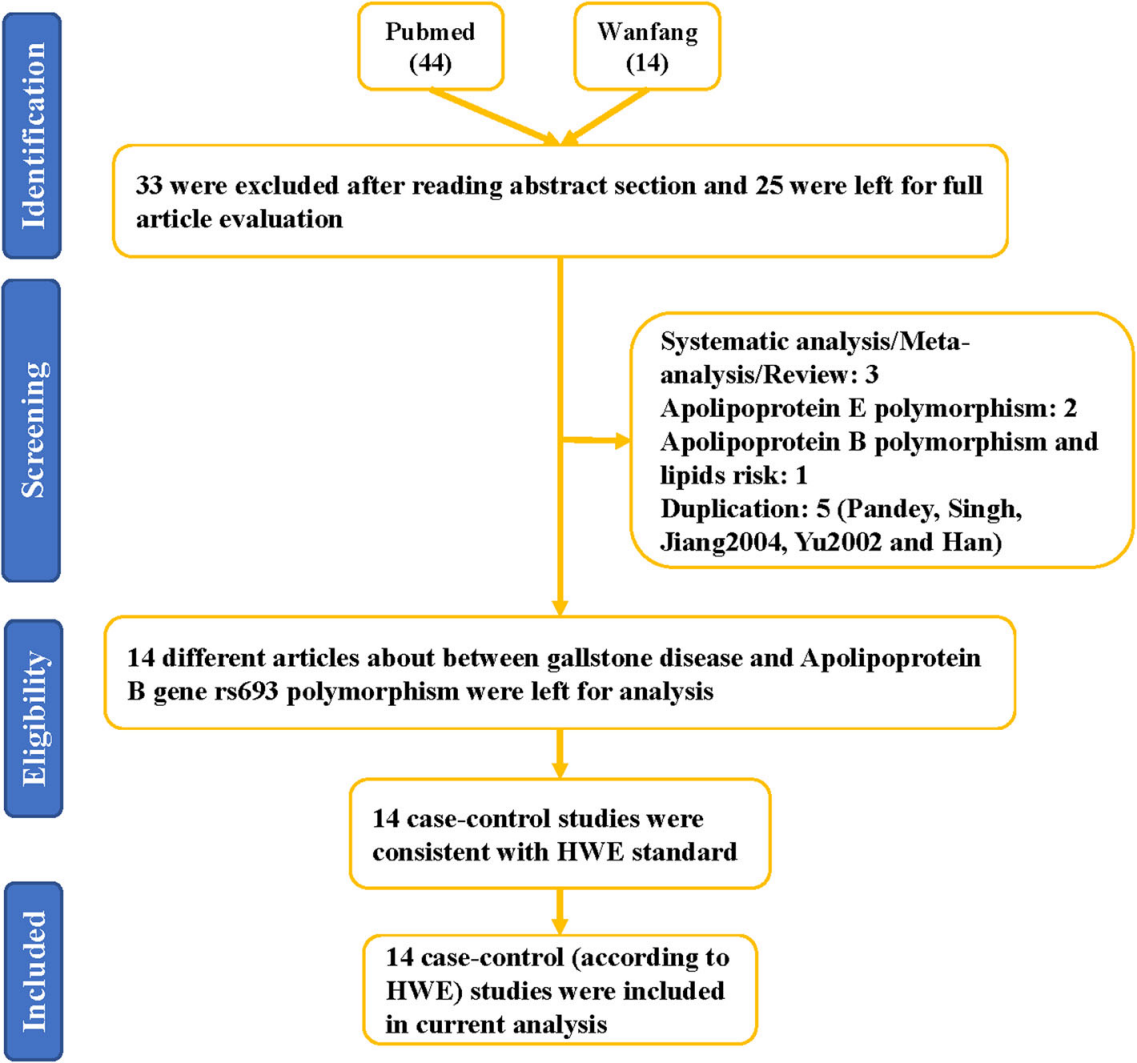
Flowchart illustrating the search strategy used to identify association studies for *APOB* gene rs693 polymorphisms and GD risk

**Fig. 2:**
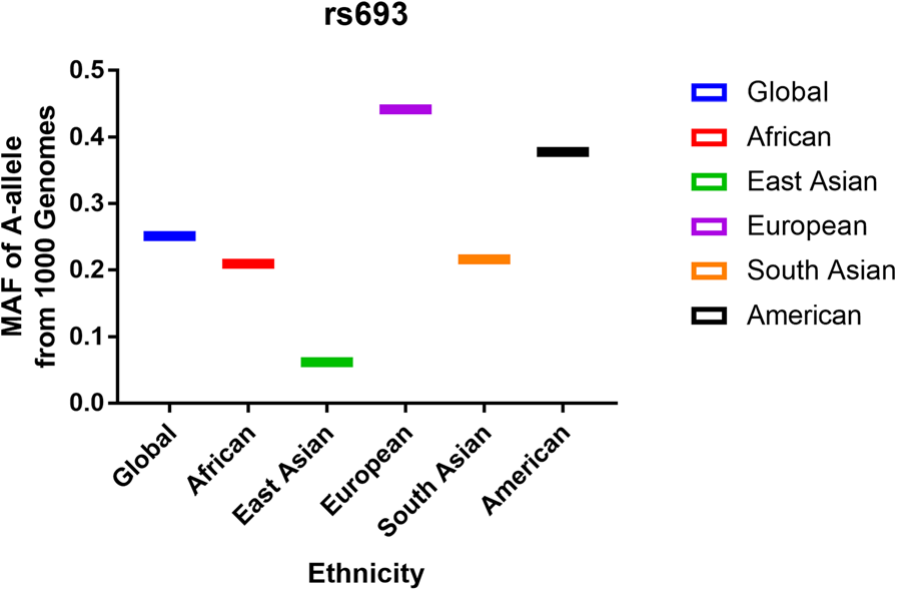
The MAF of minor-allele (mutant-allele) for *APOB* gene rs693 polymorphism from the 1000 Genomes online database and present analysis

**Fig. 3:**
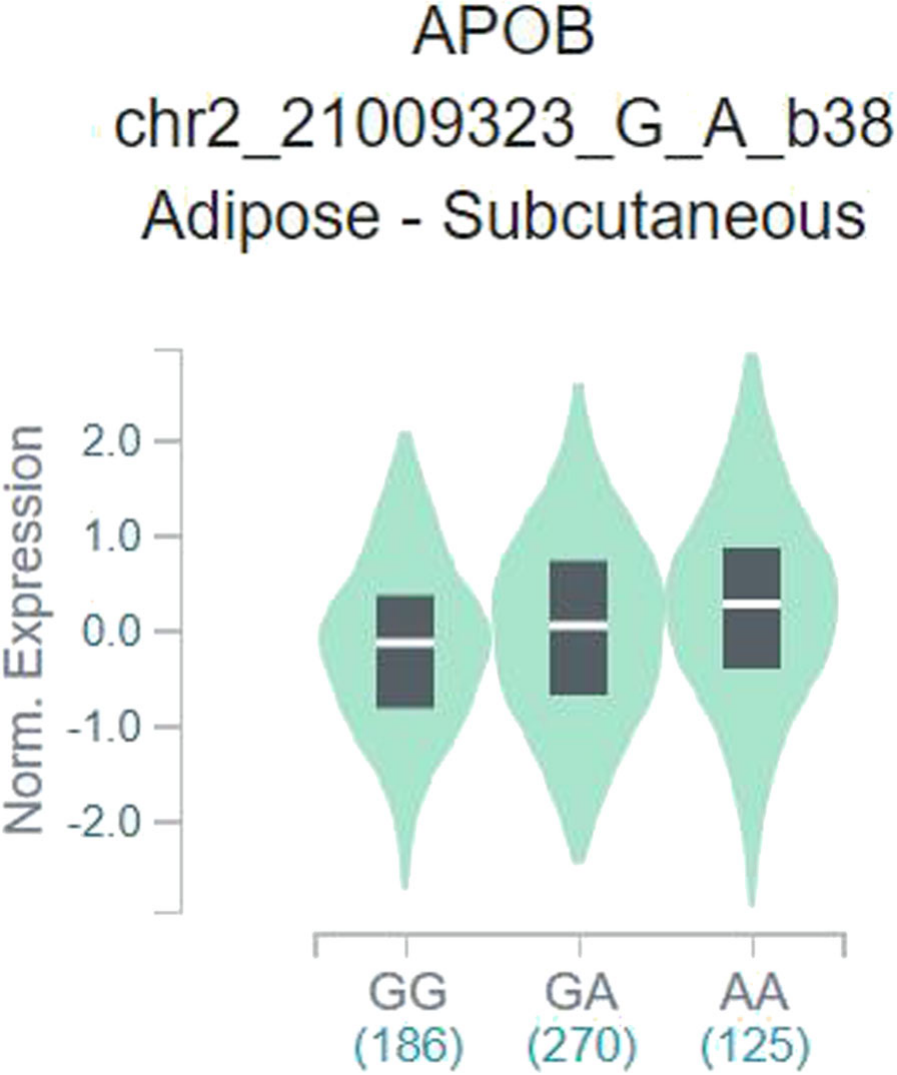
The different genotypes based on online GTEx Portal (https://www.gtexportal.org/home/)

As depicted in Fig. 1 and 58 articles were gathered from PubMed (44 titles) and Wanfang (14 titles) databases. Moreover, 33 obviously irrelevant articles were excluded after screening the titles and abstract sections. The full texts were then evaluated, and 11 additional articles were further excluded as they were duplications (5); a meta-analysis, systematic analysis, or review (2); considered other gene polymorphisms (3); and associated with the risk of another disease (1). Finally, 14 different articles [15–28] met the inclusion criteria and were included in our meta-analysis. Among these, eight were performed in China, two in Poland, one in India, one in the UK, and one in Japan. All included studies used blood samples for DNA extraction. In addition, all case-control studies about the rs693 polymorphism were consistent with the HWE in control groups (Table 1). In addition, we checked the minor allele frequency reported for the six main worldwide populations in the 1000 Genomes Browser (https://www.ncbi.nlm.nih.gov/snp/rs693) as follows: global (0.251); Europe (0.4423); East Asian (0.0615); South Asian (0.216); African (0.2095); American (0.378) (Fig. 2). In addition, we tested this polymorphism with respect to whether it influences the expression of APOB by analyzing different genotypes based on the GTEx Portal (https://www.gtexportal.org/home/). We found individuals carrying the AA genotype had higher APOB expression; however, it was determined that GG genotype-carriers might have lower expression of APOB (Fig. 3). The genotyping methods included polymerase chain reaction-restrictive fragment length polymorphism, sequencing, and TaqMan. Finally, we evaluated whether the rs693 polymorphism can influence *APOB* gene expression, and an online analysis service (https://www.gtexportal.org/home/) was applied. Results implied that individuals carrying the AA genotype might have higher APOB expression than those with the GG genotype, which suggested that the rs693 polymorphism can result in a change to the APOB protein and its functions.

**Table 1:** Characteristics of included studies in *APOB* rs693 polymorphism and gallstone disease risk

Author	Year	Country	Ethnicity	Sex	Case	Control	Case			Control			SOC	HWE	Genotype
				subgroup			AA	AG	GG	AA	AG	GG			
Dixit [16]	2008	India	Asian		206	320	6	83	117	16	127	177	PB	0.261	sequence
Juvonen [17]	1995	UK	Caucasian		76	92	9	27	40	15	42	35	HB	0.689	PCR–RFLP
Kurzawski [18]	2007	Poland	Caucasian		240	217	48	129	63	34	122	61	PB	0.076	PCR–RFLP
Rudzińska [19]	2015	Poland	Caucasian		59	58	12	30	17	12	31	15	HB	0.584	PCR–RFLP
Báez [15]	2010	Japan	Asian		119	70	13	65	41	14	31	25	HB	0.442	Taqman
Sánchez-Cuén [20]	2010	México	Caucasian		101	101	9	51	41	17	50	34	PB	0.848	PCR–RFLP
Jiang [23]	1999	China	Asian		189	442	0	39	150	0	35	407	PB	0.386	PCR–RFLP
Ji [22]	2014	China	Asian		55	65	0	3	52	0	2	63	HB	0.899	PCR–RFLP
Gu	2006	China	Asian		75	112	0	14	61	0	8	104	HB	0.659	PCR–RFLP
Tan [25]	2003	China	Asian		106	105	0	22	84	0	11	94	PB	0.571	PCR–RFLP
Yu [28]	2005	China	Asian		70	43	0	20	50	0	5	38	HB	0.685	PCR–RFLP
Suo [24]	1999	China	Asian		101	50	0	24	77	0	4	46	HB	0.768	PCR–RFLP
Wei [26]	2001	China	Asian		106	64	1	25	80	0	6	58	HB	0.693	PCR–RFLP
Yang [27]	2009	China	Asian		80	55	0	25	55	0	7	48	HB	0.614	PCR–RFLP
Sex subgroup															
Dixit [16]	2008	India	Asian	Male	64	115	3	24	37	7	46	62			
Dixit [16]	2008	India	Asian	Female	142	205	3	59	80	9	81	115			
Rudzińska [19]	2015	Poland	Caucasian	Female	59	58	12	30	17	12	31	15			
Wei [26]	2001	China	Asian	Male	37	27	1	10	26	0	3	24			
Wei [26]	2001	China	Asian	Female	69	37	0	15	54	0	3	34			
Jiang [23]	1999	China	Asian	Male	114	299	0	19	95	0	24	275			
Jiang [23]	1999	China	Asian	Female	75	143	0	20	55	0	11	132

### Quantitative synthesis

**Fig. 4:**
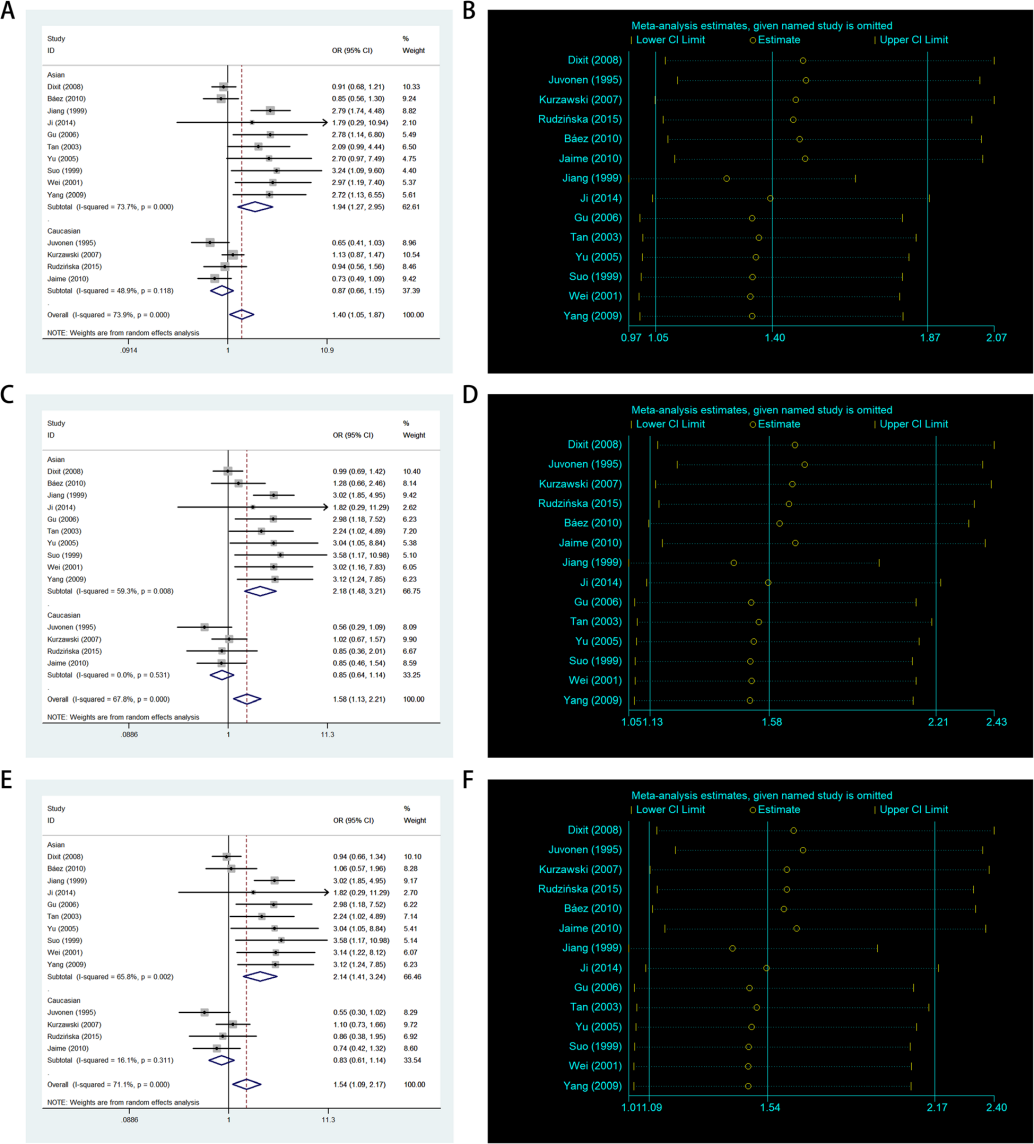
A-allele frequencies for the *APOB* gene rs693 polymorphism among cases/controls stratified by ethnicity. **a** A-allele vs. G-allele model; **c** AG vs. GG model; **e** AA + AG vs. GG model. Sensitivity analysis between *APOB* gene rs693 polymorphism and GD risk. **b** A-allele vs. G-allele model; **d** AG vs. GG model; **f** AA + AG vs. GG model

**Fig. 5:**
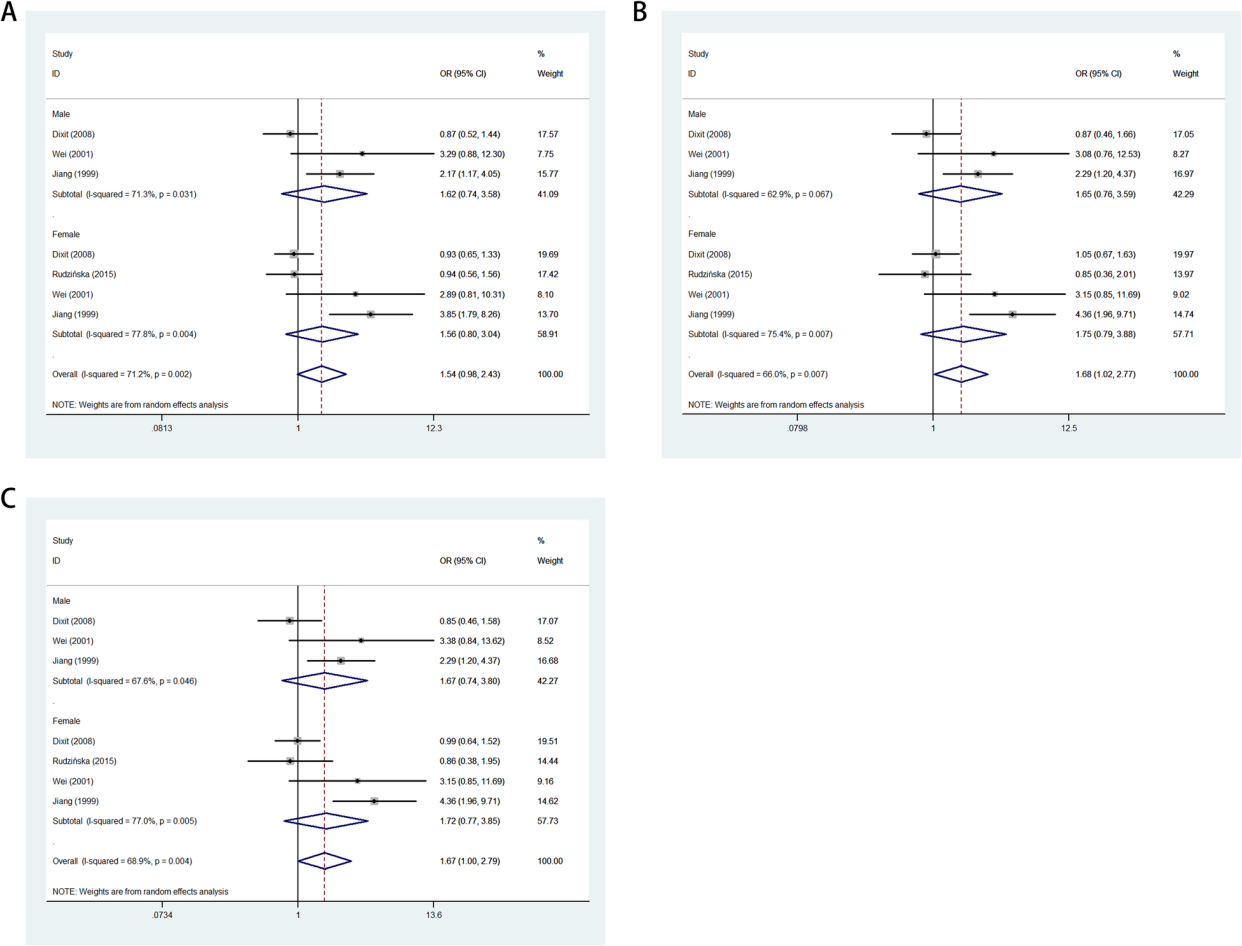
Forest plot of GD risk associated with *APOB* gene rs693 polymorphism by sex subgroup. **a** A-allele vs. G-allele model; **c** AG vs. GG model; **e** AA + AG vs. GG model

In the entire analysis, increased associations were observed in the three genetic models (allelic contrast: OR: 1.40, 95 % CI: 1.05–1.87, *P*_heterogeneity_ < 0.001, *P* = 0.023, *I*^2^ = 73.9 %; heterozygote comparison: OR: 1.58, 95 % CI: 1.13–2.21, *P*_heterogeneity_ < 0.001, *P* = 0.007, *I*^2^ = 67.8 %; dominant model: OR: 1.54, 95 % CI: 1.09–2.17, *P* < 0.001 for heterogeneity, *P* = 0.014, *I*^2^ = 71.1 %). In subgroup analysis by ethnicity, based on different frequencies of races, there were also increased associations between this polymorphism and GD in Asians, but not in Europeans, in all models (A-allele vs. G-allele: OR: 1.94, 95 % CI: 1.27–2.95, *P*_heterogeneity_ < 0.001, *P* = 0.002, *I*^2^ = 73.7 %, Fig. 4 A; AG vs. GG: OR = 2.18, 95 %CI = 1.48–3.21, *P*_heterogeneity_ = 0.008, P < 0.001, *I*^2^ = 59.3.9 %, Fig. 4 C; AA + AG vs. GG: OR: 2.41, 95 % CI: 1.41–3.24, *P*_heterogeneity_ = 0.002, *P* < 0.001, *I*^2^ = 65.8 %, Fig. 4E). In addition, regular analysis with source of control also showed a significant trend for this SNP in HB rather than PB studies (such as A-allele vs. G-allele: OR: 1.63, 95 % CI: 1.07–2.49, *P*_heterogeneity_ < 0.001, *P* = 0.023, *I*^2^ = 70.0 %). Finally, different sexes had a different incidence, and we tried to analyze this relationship in the sex subgroup as to whether significant associations exist in our analysis, but unfortunately, no significant association was found both for males and females in the three models (Fig. 5 A–C; Table 2).

**Table 2:** Results of the meta-analysis on *APOB* rs693 polymorphism and gallstone disease risk in total and types of subgroups

Variables	N	Case/Control	Allelic contrast	Heterozygote comparison	Dominant model
			OR(95 %CI) *P*_h_*P* I–squared	OR(95 %CI) *P*_h_*P* I–squared	OR(95 %CI) *P*_h_*P* I–squared
Total	14	1583/1794	1.40(1.05–1.87)0.000 0.023 73.9 %	1.58(1.13–2.21)0.000 0.007 67.8 %	1.54(1.09–2.17)0.000 0.014 71.1 %
Ethnicity					
Asian	10	1107/1326	1.94(1.27–2.95)0.000 0.002 73.7 %	2.18(1.48–3.21)0.008 0.000 59.3 %	2.14(1.41–3.24)0.002 0.000 65.8 %
European	4	476/468	0.92(0.77–1.11)0.118 0.382 48.9 %	0.85(0.64–1.14)0.531 0.285 0.0 %	0.85(0.64–1.12)0.311 0.243 16.1 %
China	8	782/936	2.68(2.01–3.58)0.997 0.000 0.0&	2.90(2.15–3.92)0.997 0.000 0.0 %	2.92(2.16–3.94)0.996 0.000 0.0 %
Not–China	6	801/858	0.91(0.79–1.05)0.307 0.200 16.5 %	0.94(0.76–1.16)0.617 0.557 0.0 %	0.90(0.73–1.11)0.540 0.315 0.0 %
SOC					
HB	9	741/609	1.63(1.07–2.49)0.000 0.023 70.0 %	1.83(1.18–2.84)0.009 0.007 59.1 %	1.79(1.13–2.84)0.002 0.014 64.9 %
PB	5	842/1185	1.28(0.84–1.94)0.000 0.256 82.9 %	1.39(0.85–2.25)0.001 0.187 78.3 %	1.35(0.82–2.23)0.000 0.232 80.5 %
Sex subgroup					
Male	3	215/441	1.62(0.74–3.58)0.031 0.231 71.3 %	1.65(0.76–3.59)0.067 0.208 62.9 %	1.67(0.74–3.80)0.046 0.219 67.6 %
Female	4	345/443	1.56(0.80–3.04)0.004 0.192 77.8 %	1.75(0.79–3.88)0.007 0.167 75.4 %	1.72(0.77–3.85)0.005 0.188 77.0 %

*P*_h_: value of *Q*-test for heterogeneity test; *P*: *Z*-test for the statistical significance of the OR

### Bias diagnosis for publication and sensitivity analysis

**Fig. 6:**
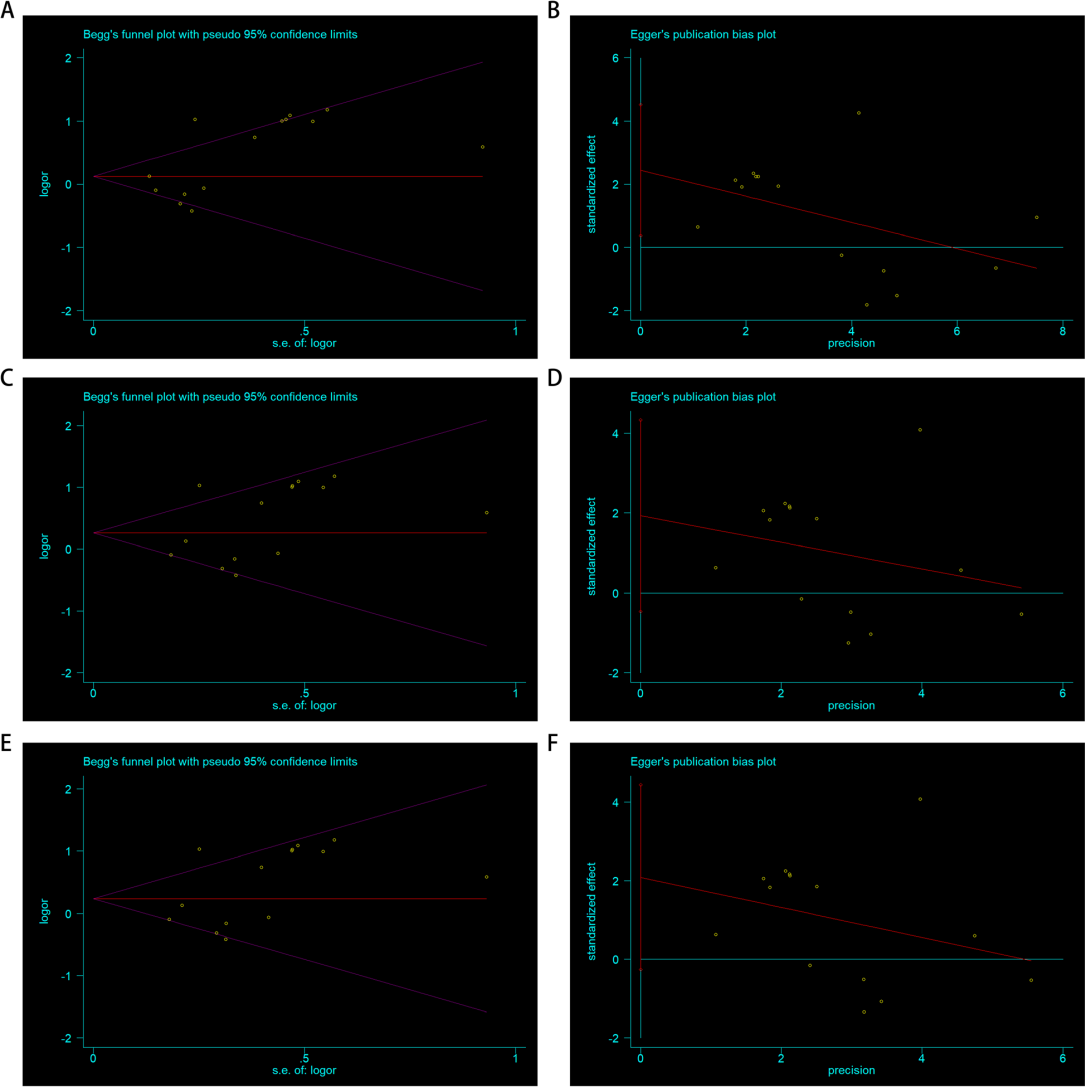
Begg’s funnel plot for publication bias test. **a** A-allele vs. G-allele model; **c** AG vs. GG model; **e** AA + AG vs. GG model. Each point represents a separate study for the indicated association. Log [OR], natural logarithm of OR. Horizontal line, mean effect size. Egger’s publication bias plot. **b** A-allele vs. G-allele model; **d** AG vs. GG model; **f** AA + AG vs. GG model

The publication bias was evaluated by using both Begg’s funnel plot and Egger’s test. At the beginning, the shape of the funnel plots seemed asymmetrical for the allele comparison of rs693 obtained using Begg’s test, suggesting that no publication bias existed. Then, Egger’s test was applied to provide statistical evidence of funnel plot symmetry. As a result, no obvious evidence of publication bias was observed (A-allele vs. G-allele: t = 2.57, *P* = 0.024 for Egger’s test; z = 1.75, *P* = 0.08 for Begg’s test; Fig. 6 A,B; Table 3). To exclude studies that might influence the power and stability of the entire study, we applied sensitivity analysis; finally, no sensitive case-control studies were found for this SNP in the three models (Fig. 4B,D,F).

**Table 3:** Publication bias tests (Begg’s funnel plot and Egger’s test for publication bias test) for *APOB* rs693 polymorphism

Egger’s test	Coefficient	Standard error	t	*P* value	95 %CI of intercept	Begg’s test	*P* value
Genetic type						z	
A–allele vs. G–allele	2.445	0.949	2.57	0.024	(0.375–4.514)	1.75	0.08
AG vs. GG	1.932	1.100	1.76	0.104	(–0.464– 4.329)	1.75	0.08
AA + AG vs. GG	2.087	1.080	1.93	0.077	(–0.266– 4.441)	1.75	0.08

### Meta‐regression

**Fig. 7:**
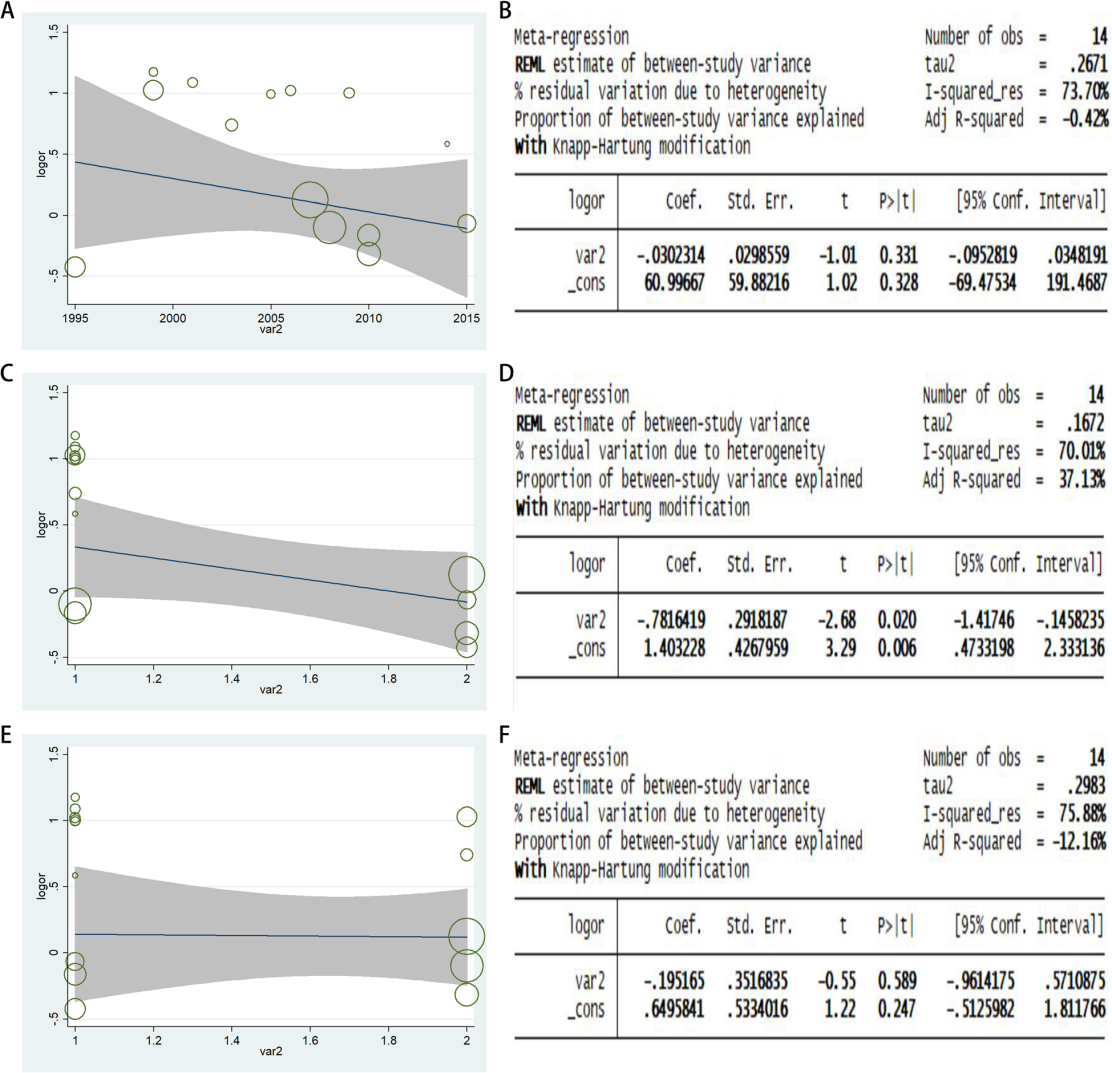
Random-effect meta-regression of log odds ratio versus publication year (**a**), ethnicity (**b**), source of control (**c**) respectively in GD

This analysis showed only a significant relationship for the allele model (A-allele vs. G-allele) for ethnicity with a regression coefficient of 0.006, rather than for the publication year and source of control subgroups, which means that the heterogeneity of the rs693 polymorphism in AF might be from the subgroup of ethnicity (Fig. 7 A–F).

### Gene–gene Network Diagram and interaction based on Online Website

**Fig. 8:**
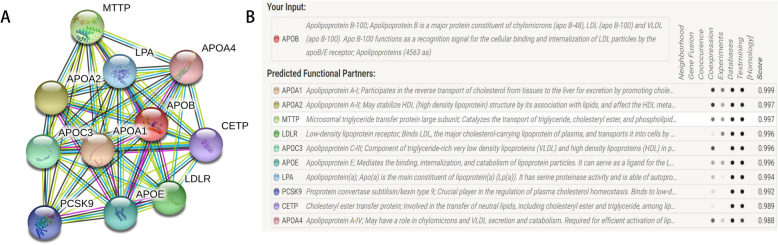
Human *APOB* interactions network with other genes obtained from String server. At least 10 genes have been indicated to correlate with *APOB* gene. *APOA1*: apolipoprotein A-I; *APOA2*: apolipoprotein A-II; *MTTP*: microsomal triglyceride transfer protein large subunit; *LDLR*: low-density lipoprotein receptor; *APOC3*: apolipoprotein C-III; *APOE*: apolipoprotein E; *LPA*: apolipoprotein (**a**); *PCSK9*: proprotein convertase subtilish/kexin type 9; *CETP*: cholesteryl ester transfer protein; *APOA4*: apolipoprotein A-IV

The String online server indicated that the *APOB* gene interacts with numerous genes. The gene–gene interaction network has been illustrated in Fig. 8.

## Discussion

GD is the most common disorder of the biliary system worldwide. The disease is generally non-life-threatening; however, the quality of life for patients is affected by upper right abdominal pain with an increased incidence of nausea, vomiting, and feelings of fullness after meals [40]. The incidence of GD has increased rapidly by nearly 2-fold every 10 years based on diet changes, widespread type-B ultrasound application, the concept of physical examinations, and other factors. Hence, further exploration of potential risk factors (besides common factors, such as pregnancy, obesity, metabolic syndrome, bariatric surgery, and ileal resection [41]) of GD should be conducted. Gu et al. carried out an observational study, suggesting that alanine transaminase activity, total standard bicarbonate, TG, and low density lipoprotein levels might be associated with the risk of GD [42].

To date, multiple genes have been shown to be associated with increased GD risk, such as ATP binding cassette subfamily G member 8, mucin-like protocadherin, and apolipoprotein E [43–45]. In addition, more and more studies have indicated that the *APOB* rs693 polymorphism might be associated with GD risk. Due to the limited number of samples used for each study, the conclusion for every study might not be credible. Dixit et al. included 214 patients with GD and 322 healthy controls and suggested that the rs693 polymorphism might not be related to GD risk[16]. In addition, Baez et al. enrolled 110 patients with GD and 70 healthy controls and showed that the rs693 variant is involved in gallstone formation and GD risk[15]. It is necessary to combine all previous studies and increase the sample size, and our aim was to obtain a comprehensive and convincing conclusion about the association between the rs693 polymorphism and GD susceptibility.

Thus, it was necessary to analyze the association between the rs693 polymorphism and GD risk using a meta-analysis method. After searching through the main database, 14 different case-control studies were identified, including 1583 cases of GD and 1794 controls. The main result of the current study is that the rs693 polymorphism is a risk factor for GD for all patients, especially in the Asian population (Chinese), which might offer a reference for early detection, prevention, and treatment. Because the incidence for GD between males and females is different, we tried to analyze whether the rs693 polymorphism differed between the two sexes; however, the analysis did not produce any positive results, which might be due to the samples.

We found publication bias in the A-allele vs. G-allele model, which might affect the strength and credibility of our conclusion. Here, we have discussed some possible reasons for this. According to the composition of the GD, it can be categorized as follows: cholesterol stones, pigment stones, and mixed stones, of which cholesterol stones are the most common; and based on the site of occurrence, GD can be divided into extrahepatic bile duct stones and hepatolithiasis, of which gallstones account for approximately 50 % of all stones. In the studies included presently, only one study indicated cholesterol stone; meta-regression was applied and showed heterogeneity. Moreover, publication bias might originate from ethnicity, because most studies were from Asian populations, especially from China, and only two studies were from Europe.

It is well known that the development of GD is complex and multi-factorial. Focusing on only one gene or one polymorphism might create a bias. Thus, we attempted to detect some related genes associated with *APOB* based on the online String server. The 10 most probable genes are shown in the network around the *APOB* gene. Among them, six are of the apolipoprotein family (subtypes), and the first related genes are *APOA1* and *APOA2*. Dixit et al. confirmed that the *APOA1* 75G/A polymorphism is associated with GD and showed sex-specific differences. However, the *APOC3* Sstl polymorphism was not found to be a factor for GD susceptibility [46]. Sarac et al. reported that increased leptin levels are associated with high LPA and APOB levels; however, in contrast, decreased APOA1 levels are found in patients with cholelithiasis [47]. Li et al. conducted meta-analyses and found insufficient evidence of an association between the *APOE* E4 polymorphism and GD risk [45]. Castro et al. suggested increased hepatic MTTP activity and bile acid synthesis in patients with GD [48]. Stender et al. found that a *PCSK9* genetic variant is associated with LDL-C level and influences the formation of GD [49]. In summary, we should thoroughly explore these partners of the *APOB* gene, as well as gene–gene interactions, in the development of GD in future studies. In addition, we tried to review the variants that are in linkage with the rs693 variant and identify probable exonic and functional variants as rs693 is a synonymous variant. Just only one paper reported by Xiao et al. was found about ischemic stroke not GD disease. They found that two blocks in *APOB* constructed by Block 1 (rs1042034, rs676210, rs693, rs673548) and Block 2 (rs3791981, rs679899) in chromosome 2 with linkage disequilibrium [50].

There are some other limitations that should be addressed as well. First, further studies should focus on mixed and African populations, which were not represented in the current analysis. Second, because GD is a multi-factorial disease, gene–gene and gene–environment interactions should be considered and assessed. It is possible that specific environmental and lifestyle factors influence the associations between the *APOB* rs693 polymorphism and GD, including age, sex, diet, diabetes, smoking, familial history, surgical history, and hypertension. Third, whether patients with GD have other complications, such as liver dysfunction, dyslipidemia, and a history of GI obstruction was not reported in the included studies. Further comprehensive studies should include such information. Fourth, the type of stone composition was not distinguished, which should be analyzed separately and can result in more accurate assessments for prediction and treatment.

## Conclusions

Our present meta-analysis suggests that the *APOB* rs693 polymorphism might be a powerful predictor of GD risk, which can serve as a detection method in clinics to provide early identification and caution patients with GD.

## Data Availability

All data generated or analyzed in this study are included in this published article and its supplementary information files.

## Abbreviations

GD: Gallbladder disease
APOB: Apolipoprotein B
HWE: Hardy–Weinberg equilibrium
OR: Odds ratio
95%CI: 95% Confidence interval
